# Investigating the effects of population density of residence and rural/urban classification on rate of influenza‐like illness symptoms in England and Wales

**DOI:** 10.1111/irv.13032

**Published:** 2022-08-03

**Authors:** Louis Tunnicliffe, Charlotte Warren‐Gash

**Affiliations:** ^1^ Department of Non‐communicable Disease Epidemiology, Faculty of Epidemiology and Population Health London School of Hygiene and Tropical Medicine London UK

**Keywords:** influenza, online community‐based cohort, population density, rural/urban living

## Abstract

**Background:**

Better understanding of risk factors for influenza could help improve seasonal and pandemic planning. There is a dearth of literature on area‐level risk factors such as population density and rural/urban living.

**Methods:**

We used data from Flusurvey, an online community‐based cohort that records influenza events. The study outcome was symptoms of influenza‐like illness (ILI). Multivariable Poisson regression analysis was used to explore associations of both population density and rural/urban status with rate of ILI symptoms and whether these effects differed by vaccination status.

**Results:**

Of the 6177 study participants, the median age was 45 (IQR 32–57), 65.73% were female, and 66% reported at least one episode of ILI symptoms between 2011 and 2016. We found no evidence to suggest that the rate of ILI symptoms was higher in the medium [RR 1.02 (95% CI 0.95–1.09)] or high [RR 1.02 (95% CI 0.96–1.09)] population density group versus the low population density group. This was the same for the effect of urban living [RR 0.96 (95% CI 0.90–1.03)] versus rural living on symptom rate. There was weak evidence to suggest that the ILI symptom rate was lower in urban areas compared with rural areas among unvaccinated individuals only [RR 0.90 (95% CI 0.83–0.99)], whereas no difference was seen among vaccinated individuals [1.04 (95% CI 0.94–1.16)].

**Conclusions:**

Although neither population density nor rural/urban status was associated with ILI symptom rate in this community cohort, future research that incorporates activity and contact patterns will help to elucidate this relationship further.

## 1 INTRODUCTION

Influenza virus results in a major healthcare and economic burden within the United Kingdom. In England alone, it was estimated to be responsible for 26,408 deaths during the 2017–2018 influenza season.^1^ Influenza is also frequently responsible workplace absenteeism, general practitioner (GP) consultations and hospital admissions, which are detrimental to the UK economically and puts strain on the National Health Service (NHS).^1^

Household overcrowding is well known to lead to increased respiratory virus transmission and has been associated with deaths during influenza pandemics.^2^ However less is known about the effect of area level factors such as population density and rural/urban living on influenza transmission.

The few observational studies that investigated associations between population density and influenza have conflicting outcomes and methodological limitations. One recent US ecological study^3^ found that more densely populated US cities or areas (by ZIP code) had higher influenza‐like illness (ILI) cases than less densely populated cities. However, this study only included cases from hospital visits and hence may have only included more severe cases because it required the individual to seek healthcare. A retrospective ecological analysis of the relationship between mortality and population density in the United States undertaken during the 1918 influenza pandemic^4^ also showed a positive association between mortality and increasing levels of population density. However, two other ecological studies^5^, ^6^ showed opposite results. One study found that mortality was higher in rural areas with lower population density during the 1918 pandemic in England and Wales.^5^ Another study conducted on the 1918 found no link between mortality and population density in Japan.^6^

The only previous individual level study was a cross‐sectional Taiwanese study conducted during the 2009–2010 H1N1 pandemic.^7^ It found that more densely populated areas of Taiwan were associated with extended epidemics.

There is a clear need for individual‐level research with objective case determination among not just hospital admissions but across entire communities. Evidence of a relationship could allow influenza control measures to be targeted to specific high‐risk areas to help to address health inequalities in respiratory‐transmitted infectious disease burden. Knowledge about the spread of influenza in relation to population density could be useful when predicting the progress of epidemics.

The aim of the present study was to investigate the effect of population density of residence and rural/urban categorisation on the incidence of influenza‐like illness symptoms.

## 2 METHODS

### 2.1 Study design and data collection

This study is a prospective online community cohort study that utilised data obtained from Flusurvey. Flusurvey is based in nine different countries and involves participants submitting weekly forms for active surveillance of the presence and absence of ILI symptoms.^8^ The present study used data from the UK's influenza survey database. Flusurvey was advertised on various media platforms,^8^ and anyone could sign up to participate. At enrolment, participants' baseline characteristics (which can be seen in Table 1) were surveyed; they were then given weekly email prompts to complete a symptoms questionnaire.^8^, ^9^

**TABLE 1:** Demographic characteristics of the cohort

Variable	Category (England + Wales percentage distribution^a^)	2011/2012	2012/2013	2013/2014	2014/2015	2015/2016	2016/2017
Number of individuals	Overall, *n* = 6177	1403	2650	2581	2794	2016	2024

Risk factors collected at the individual level		*n* (% of individuals)					
Sex	Female (50.8)	853 (60.8)	1672 (63.1)	1683 (65.2)	1837 (65.8)	1310 (65.0)	1284 (63.4)
	Male (49.2)	550 (39.2)	978 (36.9)	898 (34.8)	957 (34.3)	706 (35.0)	740 (36.6)
Age group (years)	0–16 (20.2)	79 (5.6)	137 (5.2)	214 (8.3)	162 (5.8)	95 (4.7)	85 (4.2)
	17–25 (10.8)	84 (6.0)	143 (5.4)	148 (5.7)	104 (3.7)	58 (2.9)	45 (2.2)
	26–44 (24.8)	540 (38.5)	949 (35.8)	866 (33.6)	889 (31.8)	585 (29.0)	538 (26.6)
	45–64 (25.6)	522 (37.2)	1040 (39.3)	973 (37.7)	1158 (41.5)	893 (44.3)	915 (45.2)
	65 + (18.5)	173 (12.3)	374 (14.1)	378 (14.7)	471 (16.9)	381 (18.9)	431 (21.3)
	**Missing values**	**5 (0.4)**	**7 (0.3)**	**2 (0.1)**	**10 (0.4)**	**4 (0.2)**	**10 (0.5)**
Vaccination status	No evidence vaccination	856 (61.0)	1731 (65.3)	1620 (62.8)	1713 (61.3)	1173 (58.2)	1077 (53.2)
	Vaccinated	547 (38.9)	919 (34.7)	961 (37.2)	1081 (38.7)	843 (41.8)	947 (46.8)
Any chronic Illness	No (82.1)	1177 (83.9)	2179 (82.2)	2140 (82.9)	2277 (81.5)	1624 (80.6)	1612 (79.6)
	Yes (17.9)	226 (16.1)	471 (17.8)	441 (17.1)	517 (18.5)	392 (19.4)	412 (20.4)
Current smoker	No (80.1)	1270 (90.5)	2421 (91.4)	2409 (93.3)	2598 (93.0)	1872 (92.9)	1902 (94.0)
	Yes (19.9)	132 (9.4)	229 (8.6)	172 (6.7)	191 (6.8)	144 (7.1)	122 (6.0)
	**Missing values**	**1 (0.1)**	**0 (0.0)**	**0 (0.0)**	**5 (0.2)**	**0 (0.0)**	**0 (0.0)**
Main activity	Homemaker	45 (3.2)	120 (4.5)	85 (3.3)	106 (3.8)	78 (3.9)	75 (3.7)
	Long‐term leave	13 (0.9)	44 (1.7)	28 (1.1)	34 (1.2)	21 (1.0)	34 (1.7)
	Other	27 (1.9)	51 (1.9)	54 (2.1)	40 (1.4)	27 (1.3)	32 (1.6)
	Full‐time employment	669 (47.7)	1216 (45.9)	1146 (44.4)	1207 (43.2)	873 (43.3)	849 (42.0)
	Part‐time employment	145 (10.3)	307 (11.6)	314 (12.2)	356 (12.7)	243 (12.1)	255 (12.6)
	Retired	227 (16.2)	464 (17.5)	460 (17.8)	570 (20.4)	455 (22.6)	490 (24.2)
	School	163 (11.6)	245 (9.3)	326 (12.6)	243 (8.7)	141 (7.0)	118 (5.8)
	self‐employed	93 (6.6)	169 (6.4)	139 (5.4)	208 (7.4)	157 (7.8)	156 (7.7)
	Unemployed	21 (1.5)	34 (1.3)	29 (1.1)	30 (1.1)	21 (1.0)	15 (0.7)
Occupation	NA (e.g. in education/retired)	590 (42.1)	970 (36.6)	982 (38.1)	1031 (36.9)	750 (37.2)	765 (37.8)
	Office worker	151 (10.8)	315 (11.9)	274 (10.6)	293 (10.5)	202 (10.0)	199 (9.8)
	Manual worker + retail	45 (3.2)	105 (4.0)	96 (3.7)	112 (4.0)	87 (4.3)	85 (4.2)
	Professional	597 (42.6)	1192 (45.0)	1178 (45.6)	1281 (45.9)	922 (45.7)	941 (46.5)
	Other	20 (1.4)	68 (2.6)	51 (2.0)	77 (2.8)	55 (2.7)	34 (1.7)
Highest education level^b^	No education (23)	40 (2.9)	67 (2.5)	60 (2.3)	74 (2.7)	49 (2.4)	47 (2.3)
	Still in education	97 (6.9)	159 (6.0)	207 (8.0)	145 (5.2)	81 (4.0)	64 (3.2)
	GCSE level (29)	80 (5.7)	229 (8.6)	192 (7.4)	227 (8.1)	157 (7.8)	140 (6.9)
	A level (12)	152 (10.8)	360 (13.6)	318 (12.3)	375 (13.4)	269 (13.3)	274 (13.5)
	BSc level (23)	343 (24.5)	646 (24.4)	652 (25.3)	734 (26.3)	552 (27.4)	555 (27.4)
	MSc level	683 (48.7)	1113 (42.0)	1126 (43.6)	1218 (43.6)	898 (44.5)	921 (45.5)
	**Missing values**	**8 (0.6)**	**76 (2.9)**	**26 (1.0)**	**21 (0.8)**	**10 (0.5)**	**23 (1.1)**
Transport use	Bike	134 (9.6)	197 (7.4)	197 (7.6)	203 (7.3)	143 (7.1)	149 (7.4)
	Car	591 (42.1)	1375 (51.9)	1297 (50.3)	1489 (53.3)	1113 (55.2)	1075 (53.1)
	Public transport	398 (28.4)	696 (26.3)	701 (27.2)	700 (25.1)	477 (23.7)	503 (24.9)
	Walk	260 (18.5)	366 (13.8)	365 (14.1)	378 (13.5)	264 (13.1)	284 (14.0)
	Other/motorbike	20 (1.4)	16 (0.6)	21 (0.8)	24 (0.9)	19 (0.9)	13 (0.6)
Frequent contact with children	No	1193 (85.0)	2213 (83.5)	2082 (80.7)	2317 (82.9)	1695 (84.1)	1762 (87.1)
	Yes	210 (15.0)	437 (16.5)	499 (19.3)	477 (17.1)	321 (15.9)	262 (12.9)

Risk factors collected at the household level		*n* (% of individuals)					
Population density group	1 least dense	319 (22.7)	598 (22.6)	552 (21.4)	672 (24.1)	495 (24.6)	486 (24.0)
	2	375 (26.7)	851 (32.1)	772 (29.9)	849 (30.4)	650 (32.2)	618 (30.5)
	3 most dense	709 (50.5)	1201 (45.3)	1257 (48.7)	1273 (45.6)	871 (43.2)	920 (45.5)
Rural or urban	Rural (18.5)	222 (15.8)	409 (15.4)	367 (14.2)	473 (16.9)	345 (17.1)	333 (16.5)
	Urban (81.5)	1181 (84.2)	2241 (84.6)	2214 (85.8)	2321 (83.1)	1671 (82.9)	1691 (83.6)
Region	Midlands (18.1)	186 (13.3)	381 (14.4)	348 (13.5)	369 (13.2)	277 (13.7)	258 (12.8)
	East of England (10.4)	176 (12.5)	291 (11.0)	262 (10.2)	332 (11.9)	253 (12.6)	234 (11.6)
	London (14.6)	406 (28.9)	624 (23.6)	712 (27.6)	631 (22.6)	434 (21.5)	438 (21.6)
	North England (26.6)	225 (16.0)	475 (17.9)	465 (18.0)	564 (20.2)	400 (19.8)	423 (20.9)
	South East England (15.4)	260 (18.5)	496 (18.7)	471 (18.3)	527 (18.9)	384 (19.1)	414 (20.5)
	South West England (9.4)	105 (7.5)	263 (9.9)	229 (8.9)	258 (9.2)	188 (9.3)	184 (9.1)
	Wales (5.5)	45 (3.2)	120 (4.5)	94 (3.6)	113 (4.0)	80 (4.0)	73 (3.6)
Number in household	1 (30.6)	405 (28.9)	767 (28.9)	755 (29.3)	788 (28.2)	634 (31.5)	657 (32.5)
	2 (34.1)	445 (31.7)	858 (32.4)	818 (31.7)	1010 (36.2)	729 (36.2)	672 (33.2)
	3–4 (40.4)	416 (29.7)	790 (29.8)	789 (30.6)	802 (28.7)	529 (26.2)	561 (27.7)
	>4 (35.3)	98 (7.0)	179 (6.8)	177 (6.9)	145 (5.2)	100 (5.0)	90 (4.5)
	**Missing values**	**39 (2.8)**	**56 (2.1)**	**42 (1.6)**	**49 (1.8)**	**24 (1.2)**	**44 (2.2)**

^a^ Percentage distribution within England and Wales from census data,^11^, ^12^ except for variables ‘any chronic illness’, ‘number household’ and ‘highest education level’, which has percentage distribution across the entirety of the United Kingdom as this data could not be obtained for England and Wales.

^b^ ‘Degree’ level or higher was the category available from census data, that is, it was not specific to BSc or MSc, so there is no value for distribution of MSc in the table. The value next to BSc reflects the percentage that had ‘any’ degree level education.

### 2.2 Study population and follow‐up

The present study restricted its analysis to participants who had completed at least two surveys, resided within England or Wales and provided a valid postcode that could be linked to census data to calculate household density and rural/urban status.

The present study's follow‐up period typically started each year on the 1st of November and lasted to 1st April, that is, participants were followed up over each winter period, which incorporated times of influenza virus circulation. We followed participants over a total of six winter periods from 2011 to 2017.

### 2.3 Outcome and exposure definitions

The outcome of interest was whether an individual had symptoms of ILI. An ILI event was determined by whether participants met the European Centre for Disease Prevention and Control (ECDC) ILI definition ‘the sudden onset of symptoms and at least one of following four systemic symptoms: fever or feverishness, malaise, headache, and myalgia, and at least one of the following three respiratory symptoms: cough, sore throat, and shortness of breath’,^10^ with the exception that participants were not required to specify whether the symptoms were of sudden onset or not. Participants that experienced ILI symptoms were then asked to record the start and end date of their symptoms. Participants could record multiple episodes of ILI symptoms.

The main exposure in the present study was population density of area of residence. The secondary exposure was type of area of residence, that is, urban or rural. On enrolment participants were asked to provide their postcode district with their baseline data. Linkage with 2011 Office for National Statistics (ONS) population density by postcode district census data^11^ enabled generation of population density values for Flusurvey participants. Participants were grouped into three categories of increasing population density: low [0–2.7 persons per hectare (pph)], medium (2.7–18.7pph) and high (18.7– maximum pph). Cut‐offs for these groups were determined based on tertiles of postcode district population density within England and Wales obtained from the 2011 Census.^11^ The ONS also has 2011 census data that mapped postcode district to rural/urban classification; hence, each individual in the present study could be classified as rural/urban based on their postcode.^11^

### 2.4 Other variables and missing data

At the start of each winter period, data were collected on a number of variables including year of study, age, sex, vaccination status, occupation, smoking status, education level, main activity, chronic illness status, frequent contact with children and number of household members. Vaccination status was recorded yearly and was based on whether the individual had been vaccinated that winter. Region was regrouped from 10 categories 7 to increase power.

Some participants had missing data in records for some winter periods, for example, on variables such as age, current smoking status, highest education level and number in household. Omitting all participants with records with missing values would have led to a significant loss of power and potentially introduced selection bias. Hence, values were imputed for the purpose of the analysis. The rules for imputation were as follows:

1. Extrapolate back in time to the last record with non‐missing value.
2. Extrapolate forwards in time to the next record with non‐missing value (without overruling rule 1).

### 2.5 Descriptive analysis

All analyses were conducted using STATA 16.1 software.

The baseline characteristics (count and percentage distribution of each variable category) of the cohort at the beginning of each winter period were summarised. If the data were available, England and Wales distributions from census data^11^, ^12^ of each variable were displayed to allow comparison of the cohort to the rest of the general population. Data that had been imputed from ‘missing’ were displayed, and the number and proportion of missing values for each variable were also presented.

### 2.6 Multivariable analysis

A multivariable Poisson regression analysis with random effects was conducted using a forward modelling approach to investigate associations between the exposures and self‐reported ILI symptoms. Year of study, age, sex and occupation were selected as a priori confounders. Occupation was considered to be a proxy measure of socio‐economic status (SES), an important potential confounder that Flusurvey did not directly measure. The highest education level was not selected for this purpose because the ‘still in education’ category would have encompassed both current university students and children at school, hence meaning potential misclassification in terms of SES. Variables such as transport that were thought to be on the causal pathway (urban areas have more public transport, which is likely to lead people to having more ILI symptoms due to increased contact with people), were ruled out from being potential confounders. Models containing the exposure and outcome with a priori confounders were run. Subsequently, other potential confounders were added one at a time to the models, and if they caused the effect estimate to change by >10%, then they were included in the final model. Whether there was any introduction of collinearity due to the addition of variables into the model was also assessed by comparing the standard errors of the log rate ratios of the crude versus adjusted models.

### 2.7 Effect modification

We first stratified by vaccination status and then ran Poisson regression analysis allowing for interaction between vaccination status and the exposure variables that was also conducted to explore this possibility of effect modification; this interaction was assessed using likelihood ratio tests.

### 2.8 Sensitivity analysis

The imputation of missing values as described in Section 2.4 has the potential to introduce bias. Hence, a sensitivity analysis was conducted excluding participants with any missing data. Results between the two analyses were compared for consistency.

### 2.9 Ethical considerations

Ethical approval for Flusurvey was granted by the London School of Hygiene & Tropical Medicine (LSHTM) Research Ethics Committee (REC), application number 5530. The LSHTM REC approved secondary use of data from Flusurvey for this analysis (Ethics Reference: 22462).

## 3 RESULTS

**FIGURE 1:**
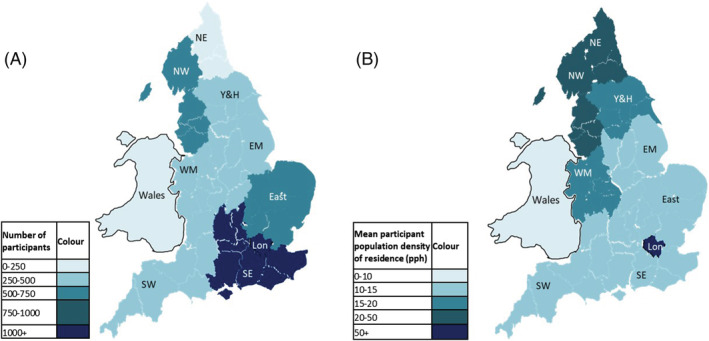
(A) The geospatial distribution of present study participants. (B) Mean population density of residence of study participants by region. Lon, London; pph, persons per hectare; NE/NW, North East/West; SW/SE, South West/East; WM/EM, West/East Midlands; Y&H, Yorkshire and Humber

Results from the descriptive analysis of the cohort can be seen in Table 1. A total of 6177 [65.73% female and median age of 45 with an interquartile range (IQR) of 32–57] individuals participated in the study across six winters, ranging from 1403 in the 2011–2012 winter to 2794 in the 2014–2015 winter. Figure 1 shows the geospatial distribution of participants. The majority of individuals were from southern England with London being the region containing the highest numbers of participants as well as being the area where participants had the highest mean population density of residence.

The results from forward modelling of the relationship between population density group and rural or urban status can be seen in Table 2. The final model consisted only of a priori (year of study, age, sex and occupation) confounders because no addition of other variables led to >10% change in the effect estimate.

**TABLE 2:** Results of multivariable Poisson regression analysis investigating the effect of population density and rural or urban living on rate of ILI symptoms

Poisson regression model	Population density group	Correlated crude incidence rate per thousand PY (95% CI)	Crude RR (95% CI)	LRT *P*‐value	Adjusted RR^a^	LRT *P*‐value
Population density group	1 Least dense	1045.47 (987.80–1106.51)	1	<0.0001	1	0.8045
	2	1065.34 (1015.08–1118.08)	1.02 (0.95–1.10)		1.02 (0.95–1.09)	
	3 Most dense	1199.50 (1152.53–1248.38)	1.15 (1.07–1.23)		1.02 (0.96–1.09)	
Rural/urban group	Rural	1085.73 (1014.94–1161.48)	1	0.2910	1	0.2684
	Urban	1128.99 (1094.97–1164.07)	1.04 (0.97–1.12)		0.96 (0.90–1.03)	

*Notes*: The LRT test for assessing whether theta = 0 in the full models gave a *P*‐value of <0.0001 in all instances. This provides strong evidence for within person clustering of ILI symptom episodes.

Abbreviations: LRT, likelihood ratio test; PY, person‐years; RR, rate ratio.

^a^ Adjusted for year of study, age, sex and occupation.

In the multivariable model for population density, the third group (most densely populated) had a rate 1.02 (95% CI 0.96–1.09) that of the baseline group (least densely populated). For the middle group the rate was 1.02 (0.95–1.10) times that of the baseline group. The likelihood ratio test for association between population density and rate of ILI symptoms gave a *P*‐value of 0.80 indicating a lack of evidence for association between population density of residence and self‐reporting ILI.

We found no evidence of association between rural/urban status and ILI symptoms (adjusted RR 0.96, 95% C.I. 0.90–1.03, *P* = 0.27).

The results of Poisson regression analysis allowing for interaction between vaccination status and each of the exposure variables can be seen in Table 3.

**TABLE 3:** ILI symptom rate ratios of population density and rural/urban groups from multivariable Poisson regression analysis of cohort stratified by vaccination group

Variables in Poisson regression model	Vaccination status	Population density group	Rate ratio (95% CI)	LRT for interaction *P*‐value
Population density group + year of study, age, sex and occupation	Vaccinated	1 (least dense)	1	0.4407
		2	1.05 (0.95–1.16)	
		3 (most dense)	1.07 (0.97–1.18)	
	Unvaccinated	1 (least dense)	1	
		2	0.99 (0.91–1.09)	
		3 (most dense)	0.99 (0.91–1.07)	
Rural or urban + year of study, age, sex and occupation	Vaccinated	Rural	1	0.0350
		Urban	1.04 (0.94–1.16)	
	Unvaccinated	Rural	1	
		Urban	0.90 (0.83–0.99)	

Abbreviation: LRT, likelihood ratio test.

There was no evidence for an interaction between influenza vaccination and population density on the rate of ILI symptoms (*P* = 0.44).

There was weak evidence (*P* = 0.04) to indicate that the ILI symptom rate was lower in urban areas compared with rural areas among unvaccinated individuals [RR 0.90 (95% CI 0.83–0.99)], whereas no difference was seen among vaccinated individuals [1.04 (95% CI 0.94–1.16)].

The sensitivity analysis provided no evidence (*P* = 0.92) to suggest that the rate of ILI symptoms was higher in the medium [RR 0.99 (95% CI 0.91–1.07)] or high [RR 1.00 (95% CI 0.92–1.07)] population density group than in the low population density group after adjusting for year of study, age, sex and occupation among individuals with no missing data. Moreover, no evidence (*P* = 0.16) was found to suggest that the rate of ILI symptoms in rural areas [RR 0.95 (95% CI 0.88–1.02)] was different to that of urban areas after the same adjustment.

## 4 DISCUSSION

Among 6177 individuals from the UK Flusurvey cohort, we found no evidence for an association between either population density or rural/urban status and rate of ILI symptoms over six winters (2011–2016). No evidence for interaction between population density and vaccination status on ILI symptom rate was found. However, there was weak evidence to suggest that the ILI symptom rate was lower in urban areas compared with rural areas among unvaccinated individuals, whereas no difference was seen among vaccinated individuals.

The results of this study do not support findings from some other observational studies that found more densely populated areas to be associated with higher influenza or ILI incidence.^3^, ^4^

This inconsistency may be because the previous studies were mainly ecological studies that cannot reliably be extrapolated to the individual level. Hence, the only comparable study was a Taiwanese cross‐sectional study, which found evidence to suggest that increased population density was associated with extended epidemics of H1N1.^7^ However, that study was limited by its cross‐sectional design and its hospital‐based nature, which meant that it only captured more severe influenza cases.

The difference in results may also reflect differences in outcome definitions. In many of the previous studies, the outcome was influenza related mortality or hospitalisation.^3^, ^4^, ^5^ This is not comparable with our symptom‐based ILI definition: Whereas influenza mortality/hospitalisation only captures the most severe influenza events, using ILI symptoms can capture milder episodes that represent the majority of cases.^13^

In addition, many of the previous studies utilised historic data, mostly from the 1918 influenza pandemic.^4^, ^5^ This combination of use of historical data, with ecological designs making it difficult to control for individual‐level confounding factors, and the fact that many previous studies were conducted during periods of pandemic, rather than seasonal, influenza circulation, is likely to account for differences in findings with the present study.

We found weak evidence that within unvaccinated individuals the rate of ILI symptoms was higher in rural areas than in urban areas, whereas within vaccinated individuals the rates were more similar. This might reflect residual differences in SES that we were unable to account for using occupation status alone. Rural areas may experience economic hardship due to low employment and residents may have less access to healthcare^14^ and other differences, for example, in patterns of comorbidities that lead to higher ILI rates in unvaccinated rural populations than unvaccinated urban populations.

The use of Flusurvey to assess the association between population density and ILI symptom incidence addresses many of the limitations of the previous studies. It is a community‐based survey, so it has less potential for selection bias when compared with studies that only included individuals that sought healthcare for ILI or died. Use of a standardised ‘ILI symptoms’ definition meant the study captured mild cases, not just those resulting in GP attendance or hospitalisation, which in turn allowed for a more representative measurement of ILI incidence within society. Flusurvey also allowed real‐time measurement of ILI symptoms, that is, using active surveillance to maximise symptom reporting compared with passive surveillance methods that rely upon people remembering and choosing to report illness. The study also allowed measurement of potential confounding variables at the individual level, something that most previous studies did not do.^9^ Moreover the cohort design allowed investigation of the temporal nature of the outcome related to exposure(s).^8^ Moreover, the present study adjusted for ‘year of study’ to account for antigenic drift between 2011 and 2017.^15^

We used participants' population density based on their postcode district (first part of postcode). However, data were not available on participants' postcode sectors, which include the first letter of the second part of a postcode, for example, for a postcode NW3 6BB, only NW3 was available. This means that true population density may have been misclassified. Although the sensitivity and specificity of the ECDC ILI case definition for detecting influenza (defined by a physician in person against gold standard RT‐PCR technique) have previously been measured to be 96.1% (95% CI 95.5–96.6) and 6.6% (95% CI 6.1–7.1) respectively,^16^ it is unclear whether the validity varies when self‐reporting is used. One study investigated the self‐reporting performance of the New Zealand Ministry of Health ILI definition, which defined ILI as fever, plus cough or sore throat. The sensitivity of this test was found to be 38.0% (95% CI 25.6–50.4) with a specificity of 67.2% (95% CI 60.6–73.8).^17^

There may also have been residual confounding by factors that are not recorded in Flusurvey such as ethnicity: Ethnicity may affect both the presentation and frequency of viral infections and likelihood of residing in areas of high population density.^18^ Other confounders may have been incompletely captured in our study: Using proxy variables such as occupation to measure SES may have led to misclassification. If information were available on participants' postcode sector, we could have measured SES more comprehensively using, for example, the Index of Multiple Deprivation (IMD).^19^

Finally, our cohort was not representative of the population of England and Wales (see Table 3), with over‐representation of females, middle aged, highly educated and people living in London. Nevertheless, although the results of this study may not be generalisable to the population of England and Wales, research has shown that internal validity is not necessarily compromised.^20^

In conclusion, results from the present study indicate that neither population density nor rural urban status based on postcode district influenced the rate of ILI symptoms. Future research that incorporates laboratory‐confirmed definitions of influenza alongside detailed postcode sector data to enable fine classification of area‐level density and deprivation, as well as individual activity and contact patterns is warranted.

## CONFLICT OF INTEREST

No conflict of interest declared.

## AUTHOR CONTRIBUTIONS

**Louis Tunnicliffe**: Study design, data management and analysis, data interpretation, drafting the manuscript. **Charlotte Warren‐Gash**: Study design, data interpretation, supervision. Both authors read and approved the final manuscript.

### PEER REVIEW

The peer review history for this article is available at https://publons.com/publon/10.1111/irv.13032.

## Data Availability

Flusurvey data collected from the Influenzanet platforms, aggregated and anonymised, are available at influenzanet.info. Interested researchers wishing to conduct scientific research can access data upon request and upon discussion with other members of the Influenzanet Scientific Committee (influenzanet.info). England and Wales census data are available online on the Office for National Statistics website (https://www.ons.gov.uk/). Postcode population density statistics are available online (https://www.nomisweb.co.uk/census/2011/ks101ew).
